# Immunodeficiency and disseminated mycobacterial infection associated with homozygous nonsense mutation of IKKβ^☆^

**DOI:** 10.1016/j.jaci.2013.12.1093

**Published:** 2014-07

**Authors:** Siobhan O. Burns, Vincent Plagnol, Beatriz Morillo Gutierrez, Daifulah Al Zahrani, James Curtis, Miguel Gaspar, Amel Hassan, Alison M. Jones, Marian Malone, Dyanne Rampling, Alex McLatchie, Rainer Doffinger, Kimberly C. Gilmour, Frances Henriquez, Adrian J. Thrasher, H. Bobby Gaspar, Sergey Nejentsev

**Affiliations:** a Institute of Immunity and Transplantation, University College London, and the Department of Immunology, Royal Free London NHS Foundation Trust, London, United Kingdom; b Great Ormond Street Hospital for Children NHS Foundation Trust, London, United Kingdom; c UCL Genetics Institute, UCL, London, United Kingdom; d Department of Pediatrics, King Abdulaziz Medical City, Jeddah, Saudi Arabia; e Department of Medicine, University of Cambridge, Cambridge, United Kingdom; f Molecular Immunology Unit, Institute of Child Health, University College London, London, United Kingdom

To the Editor:

Nuclear factor kappa B (NF-κB) signaling is known to be important for host protection against infection. For activation, proteins of the NF-κB transcription factor must be released from constitutive interaction with inhibitory IκB proteins (IκBα, IκBβ, and IκBε), which sequester NF-κB complexes in the cytoplasm. This occurs through phosphorylation and degradation of IκB proteins by the upstream IκB kinase (IKK) complex, for example, following stimulation of cell surface receptors. The IKK complex consists of 2 catalytically active kinases, IKKα and IKKβ, and the regulatory subunit IKKγ, also known as NF-κB essential modulator (NEMO). In the canonical pathway of NF-κB activation, IκBα undergoes IKKβ-dependent phosphorylation and ubiquitin-mediated degradation, liberating the NF-κB heterodimer, which then translocates to the nucleus.^1^ Mutations in 2 proteins of the NF-κB signaling pathway, NEMO and IκBα, have been described in humans and result in immunodeficiency (ID), usually associated with ectodermal dysplasia (EDA). Hypormorphic hemizygote NEMO mutations cause X-linked EDA-ID, while hypermorphic IκBα mutations lead to an autosomal-dominant EDA-ID.^2-4^ Affected individuals are susceptible to severe infections with pyogenic bacteria and mycobacteria and in some cases opportunistic and viral pathogens.^5^ In addition, an autosomal-recessive mutation in IKKα has been associated with an *in utero* lethal Cocoon syndrome characterized by multiple fetal malformations.^6^

Here, we report an 18-month-old female, second child of first-degree consanguineous parents from the Arabian Peninsula (see Fig E1, *A*, in this article's Online Repository at www.jacionline.org), who presented at age 2 months with omphalitis and delayed separation of the umbilical cord, necessitating surgical removal. At age 3 months, she developed Salmonella sepsis and subsequently suffered severe recurrent infections caused by a range of organisms including *Acinetobacter*, *Enterobacter*, *Stenotrophomonas*, and *Achromobacter* species, rotavirus, and *Candida*. Chronic diarrhea and a generalized maculopapular rash were persistent from the neonatal period. Disseminated BCGosis was diagnosed from skin and gut biopsies, and antimycobacterial treatment started at age 4 months. Her family history was significant for a brother who had died at age 1 month from *E coli* sepsis and meningitis. In addition, 3 paternal grand uncles had died in infancy with short febrile illnesses.

At the age of 18 months, our patient had conical teeth, hepatosplenomegaly, and a severe skin rash (Fig 1, *A* and *B*). This was confirmed to be persistent BCGosis on skin biopsy showing a mixed inflammatory infiltrate and possible epithelioid granuloma formation (Fig E1, *B*), with acid-fast bacilli visible and a PCR positive for DNA of *Mycobacterium tuberculosis* complex, but negative for the *esat-6* gene. Initial immunology investigations demonstrated normal proportions and numbers of naive and memory T, B, and natural killer cells, normal T-cell repertoire, and T-cell proliferation to PHA (Table I). Expression of MHC class II molecules was preserved. Neutrophil number, respiratory burst, and integrin expression were normal, as was phenotyping for monocytes and dendritic cell populations. Immunoglobulin levels had been documented to be low on repeated sampling at age 3 months (IgG 1.2 g/L, IgA < 0.05 g/L, and IgM < 0.05 g/L) and therefore immunoglobulin replacement had already been commenced. Liver function test results were normal (including aspartate transaminase, gamma-glutamyl transpeptidase, alkaline phosphatase, albumin, total protein, and total bilirubin levels). Whole blood cytokine assays on 2 occasions demonstrated severely impaired production of IFN-γ to all stimuli, absent production of IL-17, and a markedly reduced production of the proinflammatory cytokines, TNF-α and IL-6, in response to a range of Toll-like receptor ligands (Fig 1, *C*). Stimulated IL-12 production was also reduced and not significantly rescued by addition of exogenous IFN-γ. The pattern of cytokine response and clinical features were not consistent with a classical IL-12/INF-γ pathway defect but would be consistent with a defect in the NF-κB pathway.

Our patient^7^ was treated with ethambutol, rifampicin, isoniazid, and pyridoxine as well as prophylactic azithromycin, fluconazole, and intravenous immunoglobulin in view of her immunodeficiency. INF-γ treatment was added at 50 μg/m^2^ and gradually increased to 200 μg/m^2^.^7^ This was not well tolerated, and she developed ulcerating skin inflammation and increasing hepatosplenomegaly necessitating low-dose steroid treatment (0.5 mg/kg) and therefore IFN-γ treatment was withdrawn. She continued to have recurrent episodes of fever and increased inflammatory markers that most likely reflected her BCGosis. She subsequently developed a progressive respiratory distress, was admitted to intensive care for noninvasive ventilation, and, unexpectedly, died from a massive gastrointestinal and pulmonary hemorrhage after a surgical central line insertion at age 25 months.

To identify the causative mutation, we used exome sequencing. Blood sample of the patient was obtained with informed consent from the parents in accordance with the Declaration of Helsinki and with approval from the ethics committees (04/Q0501/119 and 06/Q0508/16). Library preparation, exome capture, and sequencing have been done according to the manufactures instructions. For exome target enrichment, Agilent SureSelect 50 Mb kit was used. Sequencing was done using Illumina HiSeq with 94 bp paired-end reads. In the exome data, we found 22,754 single-nucleotide variants and small insertions/deletions, including 172 very rare ones, that is, those not seen in the 1000 Genomes database (April 2012 data release) and our internal databases. Five of these variants were homozygous (see Table E1 in this article's Online Repository at www.jacionline.org). Among these we identified a homozygous nonsense mutation c.321C>A in the *IKBKB* gene that encodes the IKKβ protein, leading to a premature stop codon p.Y107X (Fig 1, *D*; see Fig E2, *A*, in this article's Online Repository at www.jacionline.org). In the exome sequence data, we found no mutations in genes encoding IκBα, NEMO, or other proteins of the NF-κB pathway. Because gene *IKBKG* that encodes NEMO was poorly covered in the exome data, we studied it by Sanger sequencing and again found no mutations. We then confirmed the *IKBKB* mutation by Sanger sequencing and showed that both parents were heterozygous carriers of this mutation (Fig E2, *B*). We then purified proteins from PBMCs and demonstrated that the patient completely lacked IKKβ expression (Fig 1, *E*). We used polyclonal rabbit anti-IKKβ antibody (Cell Signaling Technology cat no. 2678) produced by immunizing animals with a synthetic peptide corresponding to residues surrounding Leu570 of the 756 amino acid IKKβ protein. Therefore, presence of a shorter IKKβ protein that potentially could be generated after reinitiation of translation seems unlikely. Further functional experiments were not possible, because no patient-derived cell lines were available after the patient's death. Given that functional reconstitution experiments were impossible, we cannot formally prove that the IKKβ deficiency has caused the immune phenotype of the patient, although it is very likely in the absence of other candidate loss-of-function mutations. However, additional unknown modifier genetic or nongenetic factors may have affected the severity of the patient's phenotype.

Here we demonstrate that an autosomal-recessive human IKKβ deficiency has a clinical presentation, which resembles both hypermorphic IκBα and hypomorphic NEMO mutations, with a prominent susceptibility to mycobacteria and an increased frequency of other bacterial infections. No loss-of-function alleles in the *IKBKB* gene were previously found in up to 6500 healthy subjects (National Heart, Lung, and Blood Institute exome variant server, accessed May 2013), suggesting that such mutations are likely to be pathogenic. Recently, a conference abstract has been published describing a group of patients with severe combined immunodeficiency with agammaglobulinemia that were associated with a frameshift mutation in the *IKBKB* gene, which apparently led to an abolished IKKβ expression.^8^ In contrast to our patient, they had reduced numbers of natural killer cells and manifested with invasive bacterial and viral, but not mycobacterial, infections. Furthermore, no ectodermal dysplasia was reported, which distinguishes them from our patient and most patients with NEMO and IκBα mutations.^5^ The reasons for such discrepancies should be clarified when a more detailed description of those patients is published.

The IKKβ-deficient mouse embryos die because of liver degeneration and apoptosis.^9-11^ In contrast, our data suggest that humans with the IKKβ deficiency can be viable. This is potentially explained by the compensatory mechanisms involving IKKα and the noncanonical pathway of NF-κB activation that are unlikely to be affected by the IKKβ deficiency. Interestingly, while NF-κB activation in cells of the IKKβ-deficient mice was significantly impaired, it was not completely abolished, as residual signaling was mediated by IKKα.^9,10^ In the absence of IKKβ, IKKα can make homodimers, which can bind NEMO and phosphorylate IκBα, thus partially compensating for the loss of the IKKα/IKKβ heterodimers.^9,12^ We did not observe significant biochemical liver disease in our patient, which may also relate to compensatory mechanisms or result from species variation.

The severe granulomatous reaction seen in our patient may be a feature of systemic infection.^13^ However, altered keratinocyte function may additionally cause skin inflammation because mice with epidermis-specific deletion of IKKβ develop a severe TNF-mediated inflammatory skin disease.^14^ Our patient had chronic diarrhea, similar to most of the patients with the IκBα mutations. This is consistent with the importance of the NF-κB pathway for mucosal immunity, maintaining intestinal epithelia integrity and homeostasis in the gut.^15^ In line with other defects of NF-κB signaling, hypogammaglobulinemia and ectodermal dysplasia, characterized by peg teeth, were features of IKKβ deficiency in our case. The immunological phenotype was characterized by a profound defect in the production of proinflammatory cytokines despite intact development of all immune cell lineages. Although the clinical spectrum of the condition will be clarified by the identification of other affected families, the severity of immunodeficiency in our patient and failure to respond to conservative therapy suggest that bone marrow transplantation should be considered for children with complete IKKβ deficiency.

## Figures and Tables

**Fig E1:**
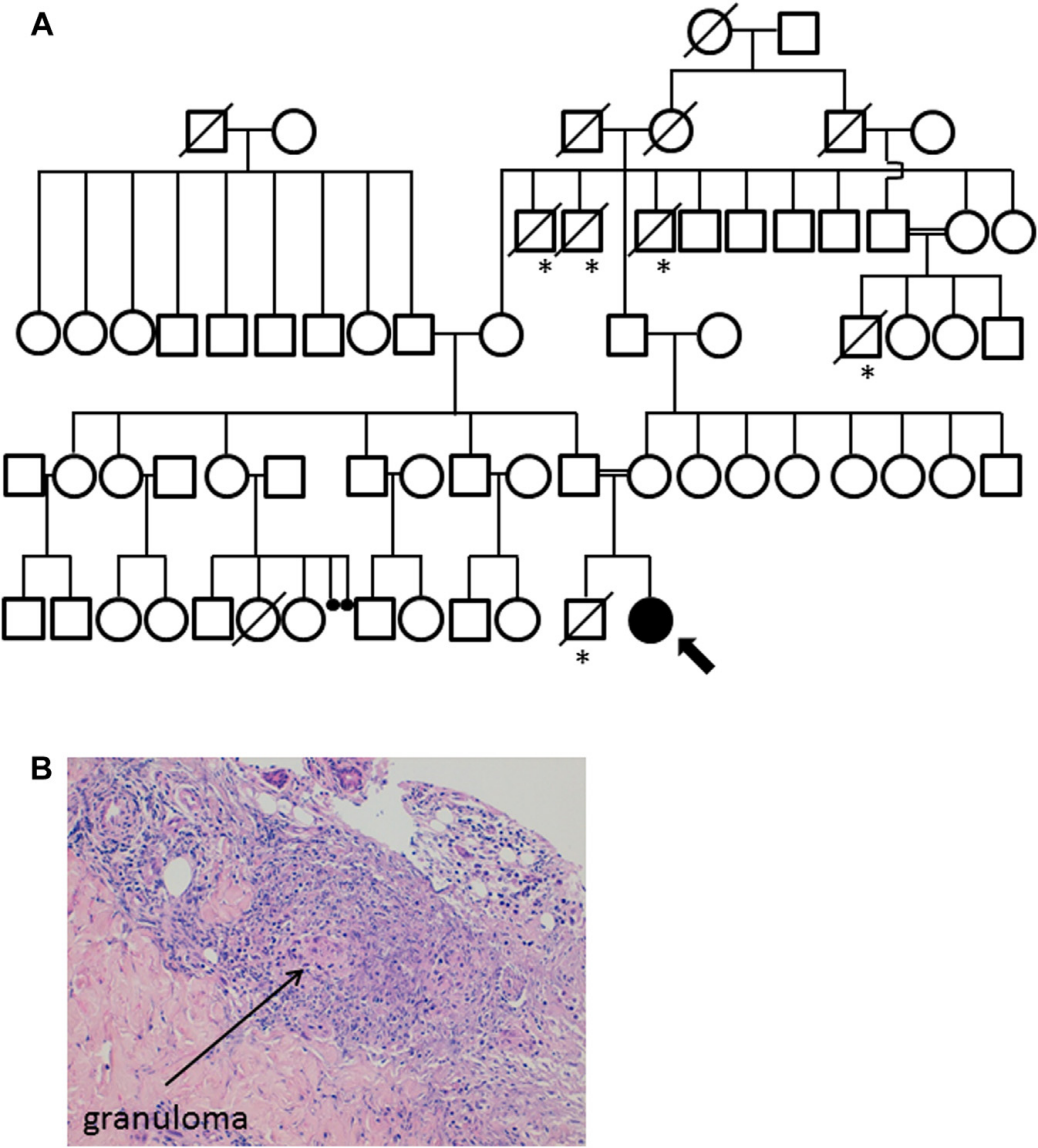
**A,** Genealogical tree for the kindred. *Arrow* highlights index case. *Asterisks* denote family members who died within the first 2 months of life with febrile illnesses. **B,** Histology showing granuloma in skin by hematoxylin and eosin staining.

**Fig E2:**
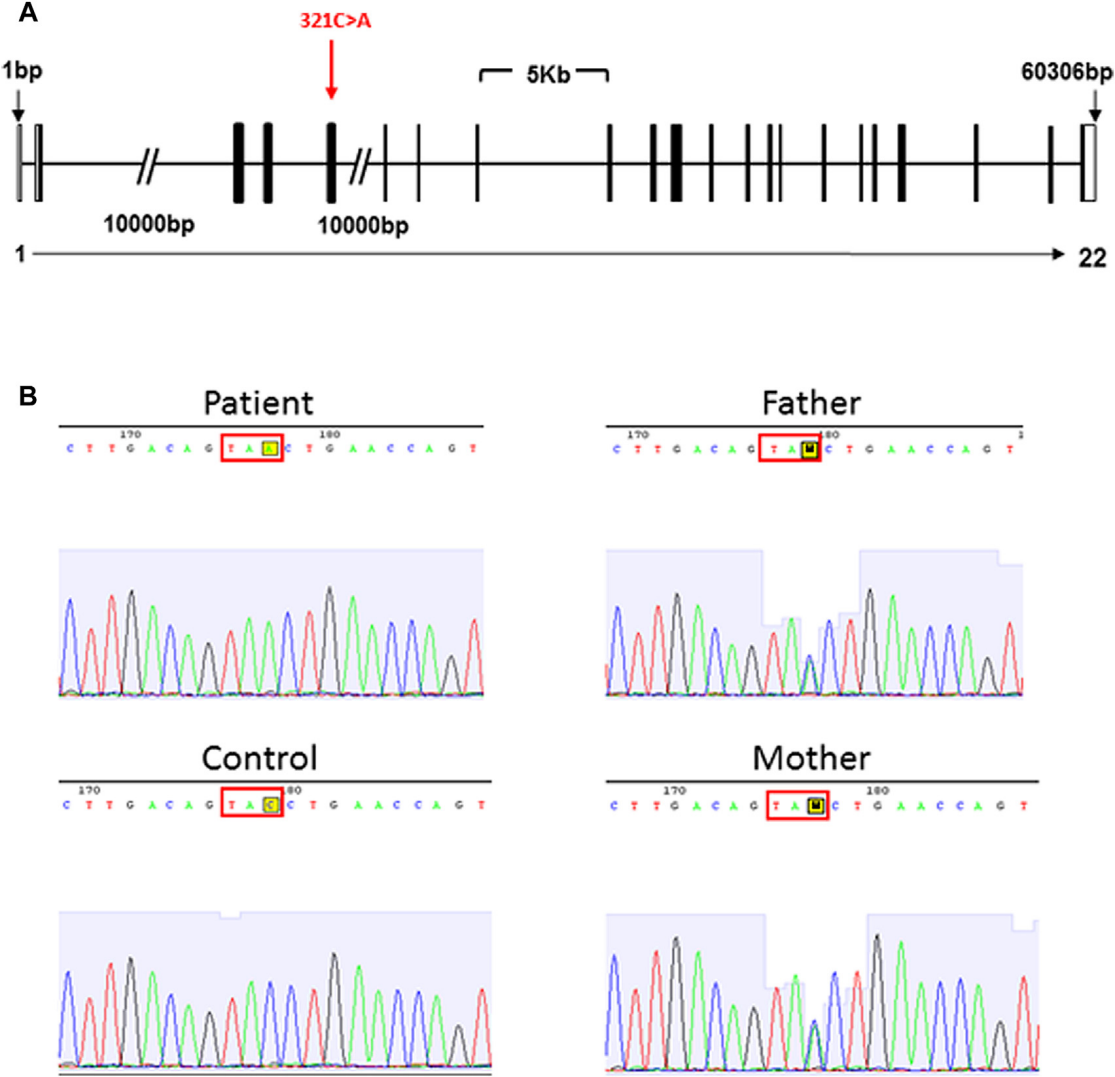
**A,** Schematic *IKBKB* gene showing the mutation identified in our family. **B,** Sequence data for the index case, parents, and healthy control showing heterozygous carriage of the c.321C>A mutation in both parents.

**Table E1:** Rare coding homozygous variants found by exome sequencing

Chr	Position	Ref/Obs	Transcript	cDNA (change)	Gene	Amino acid
1	109,276,144	—/T	ENST00000370017	c.2130_2131insT (frameshift insertion)	FNDC7 (fibronectin type III domain containing 7)	p.K710fs
1	116,280,898	C/T	ENST00000261448	c.G479A (nsSNV)	CASQ2 (calsequestrin 2)	p.R160H
1	120,168,566	G/A	ENST00000421812	c.C158T (nsSNV)	ZNF697 (zinc finger protein 697)	p.P53L
2	216,197,149	A/G	ENST00000236959	c.A733G (nsSNV)	ATIC (5-aminoimidazole-4-carboxamide ribonucleotide formyltransferase/IMP cyclohydrolase)	p.N245D
8	42,150,963	C/A	ENST00000520810	c.C321A (stopgain SNV)	IKBKB (inhibitor of kappa light polypeptide gene enhancer in B cells, kinase beta)	p.Y107X

**Fig 1:**
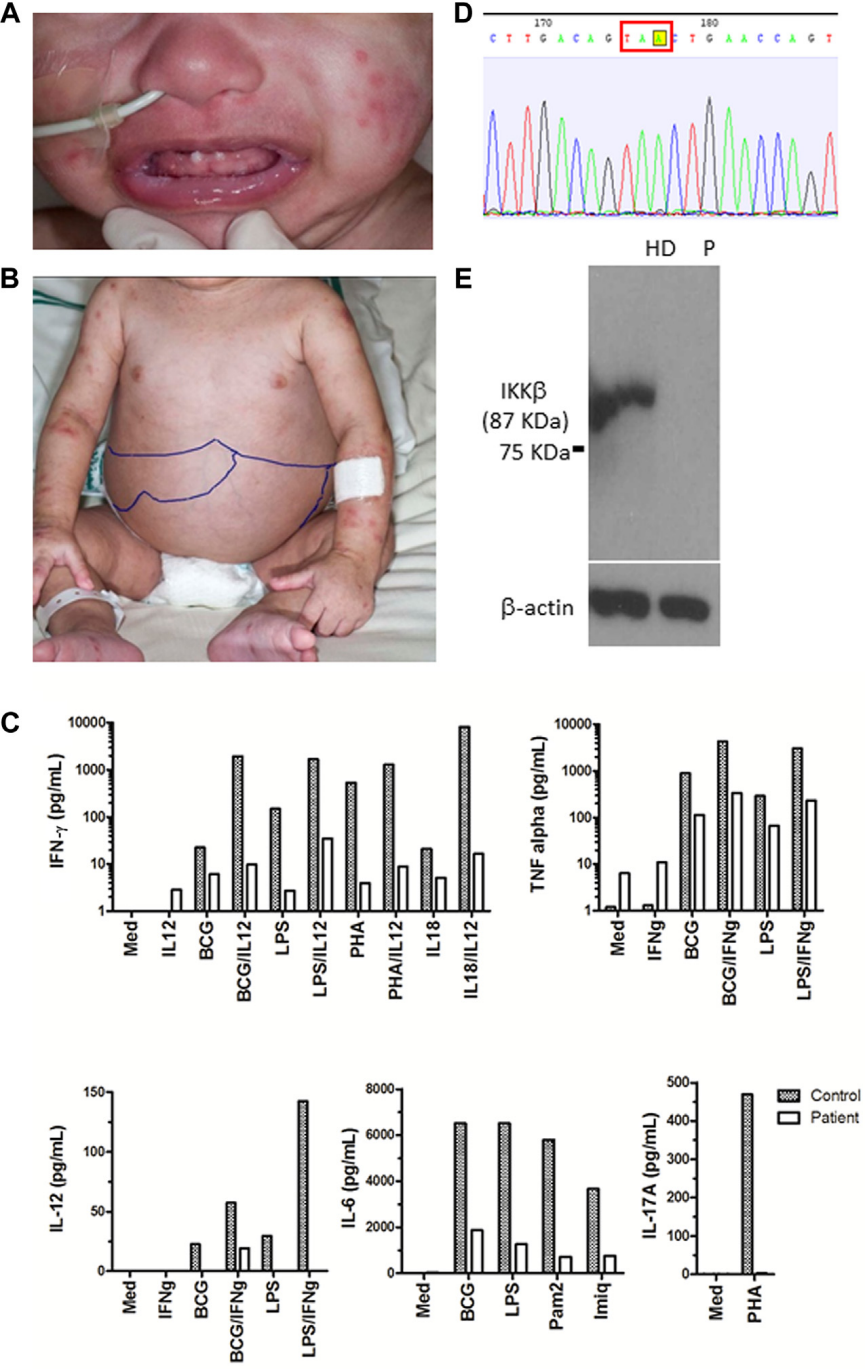
**A** and **B,** Photographs showing conical teeth, hepatosplenomegally, and widespread rash. **C,** Quantitation of cytokine release from patient and healthy control PBMCs following *in vitro* activation with various stimuli *(y-axis)*. Representative data from 1 of 2 repeat experiments are shown. **D,** Sequence data for the index case showing the homozygous c.321C>A mutation. **E,** Western blot showing that the IKKβ protein is present in the healthy donor (HD) but is absent from the patient's *(P)* PBMCs.

**Table I:** Immunology investigations

Cell type	Patient's (age 20 mo) results	Age-matched control range
White cell count	8.42 × 10^9^/L	(5.0-15.0) × 10^9^/L
Neutrophil count	3.87 × 10^9^/L	(1.0-8.5) × 10^9^/L
Lymphocyte count	4.03 × 10^9^/L	(3.0-13.5) × 10^9^/L
CD3 T cells	89.0%, 3.37 × 10^9^/L	39%-73%, (1.8-8.0) × 10^9^/L
CD19 B cells	7.0%, 0.27 × 10^9^/L	17%-41%, (0.6-3.1) × 10^9^/L
CD16^+^CD56^+^ natural killer cells	3.0%, 0.11 × 10^9^/L	3%-16%, (0.1-1.4) × 10^9^/L
CD3^+^CD4^+^ T cells	61.0%, 2.31 × 10^9^/L	25%-50%, (0.9-5.5) × 10^9^/L
CD3^+^CD8^+^ T cells	27.0%, 1.02 × 10^9^/L	11%-32%, (0.4-2.3) × 10^9^/L
Naive T cells (CD4^+^CD45RA^+^CD27^+^)	90.0%	62%-90%
Naive T cells (CD8^+^CD45RA^+^CD27^+^)	98.0%	46%-85%
Naive B cells (CD19^+^IgD^+^CD27^−^)	98.0%∗	42.6%-82.3%
Nonswitched memory B cells (CD19^+^IgD^+^CD27^+^)	1.0%∗	7.4%-32.5%
Class switched memory B cells (CD19^+^IgD^−^CD27^+^)	0.2%∗	6.5%-29.1%
Transitional B cells (CD19^+^IgM^++^CD38^++^)	6.0%∗	0.6%-3.4%
Plasmablasts (CD19^+^CD38^++^IgMwk)	0.0%∗	0.4%-3.6%
CD21 low B cells (CD19^+^CD21wkCD38wk)	2.0%	0.9%-7.6%
IgM/IgD B cells	Present	Present
IgG	6.37 g/L after IVIG	3.1-13.8 g/L
IgA	<0.23 g/L∗	0.3-1.2 g/L
IgM	3.57 g/L∗	0.5-2.2 g/L

*IVIG*, Intravenous immunoglobulin therapy.

∗ Abnormal result.

## References

[1] Oeckinghaus A., Hayden M.S., Ghosh S. (2011). Crosstalk in NF-kappaB signaling pathways. Nat Immunol.

[2] Doffinger R., Smahi A., Bessia C., Geissmann F., Feinberg J., Durandy A. (2001). X-linked anhidrotic ectodermal dysplasia with immunodeficiency is caused by impaired NF-kappaB signaling. Nat Genet.

[3] Filipe-Santos O., Bustamante J., Haverkamp M.H., Vinolo E., Ku C.L., Puel A. (2006). X-linked susceptibility to mycobacteria is caused by mutations in NEMO impairing CD40-dependent IL-12 production. J Exp Med.

[4] Courtois G., Smahi A., Reichenbach J., Doffinger R., Cancrini C., Bonnet M. (2003). A hypermorphic IkappaBalpha mutation is associated with autosomal dominant anhidrotic ectodermal dysplasia and T cell immunodeficiency. J Clin Invest.

[5] Picard C., Casanova J.L., Puel A. (2011). Infectious diseases in patients with IRAK-4, MyD88, NEMO, or IkappaBalpha deficiency. Clin Microbiol Rev.

[6] Lahtela J., Nousiainen H.O., Stefanovic V., Tallila J., Viskari H., Karikoski R. (2010). Mutant CHUK and severe fetal encasement malformation. N Engl J Med.

[7] Alangari A.A., Al-Zamil F., Al-Mazrou A., Al-Muhsen S., Boisson-Dupuis S., Awadallah S. (2011). Treatment of disseminated mycobacterial infection with high-dose IFN-gamma in a patient with IL-12Rbeta1 deficiency. Clin Dev Immunol.

[8] Pannicke U., Baumann B., Fuchs S., Rensing-Ehl K., Holzmann K., Henneke P. (2012). A novel SCID due to an IKBKB defect. J Clin Immunol.

[9] Li Q., Van A.D., Mercurio F., Lee K.F., Verma I.M. (1999). Severe liver degeneration in mice lacking the IkappaB kinase 2 gene. Science.

[10] Tanaka M., Fuentes M.E., Yamaguchi K., Durnin M.H., Dalrymple S.A., Hardy K.L. (1999). Embryonic lethality, liver degeneration, and impaired NF-kappa B activation in IKK-beta-deficient mice. Immunity.

[11] Li Z.W., Chu W., Hu Y., Delhase M., Deerinck T., Ellisman M. (1999). The IKKbeta subunit of IkappaB kinase (IKK) is essential for nuclear factor kappaB activation and prevention of apoptosis. J Exp Med.

[12] Woronicz J.D., Gao X., Cao Z., Rothe M., Goeddel D.V. (1997). IkappaB kinase-beta: NF-kappaB activation and complex formation with IkappaB kinase-alpha and NIK. Science.

[13] Samaniego S., Marcu K.B. (2013). IKKbeta in myeloid cells controls the host response to lethal and sublethal Francisella tularensis LVS infection. PLoS One.

[14] Pasparakis M., Courtois G., Hafner M., Schmidt-Supprian M., Nenci A., Toksoy A. (2002). TNF-mediated inflammatory skin disease in mice with epidermis-specific deletion of IKK2. Nature.

[15] Pasparakis M. (2012). Role of NF-kappaB in epithelial biology. Immunol Rev.

